# Biphasic Kinetics of IGF-I and IGFBP-3 in Response to Military Field Training in Brazilian Air Force Recruits

**DOI:** 10.1093/milmed/usae097

**Published:** 2024-04-30

**Authors:** Thomaz Talarico Neto, José Maurício Magraner, Higino Carlos Hahns Júnior, Leandro Ferreira, Carlos Eduardo Martinelli Júnior, Hugo Tourinho Filho

**Affiliations:** School of Physical Education and Sport of Ribeirao Preto—EEFERP/USP, University of Sao Paulo—USP; 3900 Bandeirantes Avenue—Campus da USP, Ribeirão Preto, SP 14049-900, Brazil; Brazilian Air Force Academy—AFA, Pirassununga, SP 13643-000, Brazil; School of Physical Education—FEF/UNICAMP, State University of Campinas; Cidade Universitária Zeferino Vaz—Barão Geraldo, Campinas, SP 13083-970, Brazil; School of Physical Education and Sport of Ribeirao Preto—EEFERP/USP, University of Sao Paulo—USP; 3900 Bandeirantes Avenue—Campus da USP, Ribeirão Preto, SP 14049-900, Brazil; University Center UNIFAFIBE, Bebedouro, SP 14701-070, Brazil; Faculty of Medicine of Ribeirão Preto, University of Sao Paulo—USP; 3900 Bandeirantes Avenue—Campus da USP, Ribeirão Preto, SP 14049-900, Brazil; School of Physical Education and Sport of Ribeirao Preto—EEFERP/USP, University of Sao Paulo—USP; 3900 Bandeirantes Avenue—Campus da USP, Ribeirão Preto, SP 14049-900, Brazil

## Abstract

**Introduction:**

Insulin-like growth factor type I (IGF-I) has gained considerable notoriety in military training, primarily because it is responsible for energy deficits and sensitive to an inadequate protein intake, which are situations that are commonly experienced in specific military operations. Therefore, this study aimed to assess the kinetics of IGF-I and insulin-like growth factor binding protein type 3 (IGFBP-3) in a 4-day military field training exercise.

**Materials and Methods:**

The sample comprised 12 male soldiers (21.71 ± 1.64 years). Changes were assessed at 3 times: time 1—basal (control week); time 2—after specific military field training; and time 3—1 week after the specific training (control week). Changes in body composition and serum levels of IGF-I and IGFBP-3 were observed.

**Results:**

The main finding of this study was it verified the biphasic kinetics of both IGF-I and IGFBP-3 at the 3 times observed, that is, a significant drop from time 1 (basal—IGF-I: 189 ng/mL and IGFBP-3: 4.71 mg/L) to time 2 (immediately after military training—IGF-I: 162 ng/mL and IGFBP-3: 4.08 mg/L) and a subsequent recovery of these markers, with a significant increase from time 2 (immediately after military training) to time 3 (a week after military training—IGF-I: 199 ng/mL and IGFBP-3: 4.96 mg/L).

**Conclusions:**

It can be concluded that IGF-I and IGFBP-3 levels respond quickly to the stimuli caused by military training, especially after specific field training. However, the same markers quickly return to their basal values after this type of training finishes, simply by following the daily routine of the battalion in the control weeks, with no specific intervention being necessary.

## INTRODUCTION

An elite soldier’s training should encompass several aspects. Discipline, physical fitness, and resilience (psychological and physical) are determining factors for success in their professional activities.^1^

Thus, to achieve such excellence, the training should be arduous. As mentioned earlier, specific activities are needed to test all these characteristics. Therefore, training exercises that come close to reality, such as simulated tasks or field exercises, are combined. They aim to simulate situations that soldiers might encounter during battlefield operations^2,3^—such as sleep restriction, food restriction, a high level of physical/energetic demand, psychological pressure, and pressure to successfully carry out the activities.^2,4,5^

Therefore, military field training can lead to various physiological alterations, including changes in the participants’ endocrine system; the body will try to adapt to enable an energy supply until the end of the training. To do this, it will adjust its hormonal release patterns.^5^

Recent studies have assessed the kinetics of the growth hormone and insulin-like growth factor type I (IGF-I) axis, a set of growth factors, receptors, and binding proteins that help in somatic tissue growth in various species.^6–10^ Among the hormones of this axis, IGF-I has gained considerable notoriety in military training, primarily because it is responsible for and sensitive to both energy deficit and inadequate protein intake, which are situations that are commonly experienced in specific military operations.^6,11–13^ Energy deficit is the main factor linked to IGF-I responses. In this environment, energy deficit increases because of low caloric intake and high energy expenditure from military training.^5–7^

With the help of IGF-I binding proteins, it becomes easier to understand stress in the body. Insulin-like growth factor binding protein type 3 (IGFBP-3) is the most abundant binding protein of this axis and is known to strengthen the actions of IGF-I; however, in a military training situation, because of caloric restriction, sleep deprivation, and intense physical activity, its values may drop, confirming its importance as a biomarker of training status.^5–8,11^

In military training that lasts as short as 3 to 8 days, a reduction in serum concentrations of IGF-I (total and free) as well as in its main binding protein IGFBP-3 is expected, as the body is not in ideal conditions for this hormone and its main binding protein to function efficiently. This reduction is a way for the body to protect itself from energy deficit.^6^ Changes in IGF-I/IGFBP-3 serum levels are the focus of several types of research because of the hormone’s role in amino acid absorption, protein synthesis, and protein degradation. IGF-I is essential for the development and regulation of healthy tissues, promoting cell proliferation and blocking apoptosis.^10^ Its use as a metabolic and individual health status biomarker justifies further investigation.^7^ Serum IGF-I concentrations decrease during caloric or protein restriction situations, but baseline levels are restored after refeeding.^3,6^

Military field training aims to expose the individual to stress. The short duration of the serum reductions in these biomarkers during training has no clinical implications. However, at the end of these field exercises, it is of the utmost importance to monitor the time needed to reset these parameters to their basal values, as the primary concern regarding the longevity of the career and the soldiers’ health is a possible serum reduction in these markers for a chronic period, which is harmful.^1,4,6^ In Brazil, there is only one study about the kinetics of IGF-I and IGFBP-3 in response to the field training of the country’s combat forces.^7,14^

Therefore, this study aimed to assess the kinetics of IGF-I and IGFBP-3 using 3 points in time: time 1—baseline (control week); time 2—after specific military field training; and time 3—1 week after the specific training (control week).

## METHODS

### Subjects

Twelve soldiers (19 ± 0.43 years, 1.75 ± 0.09 m, 71.46 ± 12.82 kg) participated in the study, all male, healthy, and recruited from the soldier’s training course. The logistics of the exercise did not allow for a sample greater than 12, so the researchers sent an invitation, and the first 12 who accepted the terms and conditions were included in this study. Only soldiers who were able to carry out the 4 days of military training were invited to participate in the study, having been approved by the institution’s doctors and staff. Participants who did not complete the 4-day military training would be excluded, but none of the volunteers withdrew.

### Ethical Considerations

The study protocol follows the ethical guidelines of the 1975 Declaration of Helsinki and was approved by the local Ethics Committee (n. 3.230.057) and the Brazilian Air Force Academy. All the volunteers were informed of the experimental design, benefits, and possible risks associated with the study before signing an informed consent form to participate in the study.

### Experimental Design

The data were collected in 3 different periods: time 1—baseline, carried out a week before the military field training; time 2—immediately after the military field training; and time 3—a week after the end of the 4 days of military field training.

At time 1, to characterize the sample, food diaries were kept (on 3 alternate days), body composition was recorded (weight and skinfolds—7 Skinfold Jackson & Pollock Protocol), and blood analysis was performed (IGF-I and IGFBP-3 baseline values). The soldiers also carried out the activities foreseen in the soldier’s training course routine, and all participants followed the same training routine.

Immediately after 4 days of field training, at time 2, blood and body composition (weight and skinfolds) were collected again. During these 4 days, all participants adhered to a meticulously planned schedule of activities and had equal access to identical types and quantities of food, with no differentiation between participants.

Finally, at time 3, 1 week after the end of the 4 days of military training, the soldiers underwent a blood analysis to verify the IGF-I and IGFBP-3 kinetics. In this week of recovery, the same routine activities as at time 1 were carried out. However, because of the available logistics (soldier’s training course routine), body composition analyses were not performed, and food diaries were not kept, and only blood samples were collected.

### Description of the Military Field Training

During the 4 days of military field training, the recruits underwent a planned routine with various specific exercises (marches of 5-10 km per day, with a load of approximately 14 kg and around 4 hours of sleep per night). On these 4 days, the recruits practiced activities such as long-distance marching; building shelters and traps; recognizing, identifying, and handling emergency procedures related to venomous creatures; camouflage techniques; approaching sentries in an enemy camp; compass and map reading; military hiking techniques; using/applying auditory, visual, olfactory, and tactile acuity in combat actions; emergency/evacuation procedures; obtaining food of animal and vegetable origin and preparing it in the field; techniques for obtaining water and fire; and tactical combat casualty care techniques.^14^

### IGF-I and IGFBP-3 Serum Determination

Blood samples were taken in the morning after fasting (12 hours) at 3 times: baseline (1 week before), immediately after the 4-day field exercise, and 1 week after the field exercise, using standardized procedures in accordance with the laboratory guidelines. IGF-I and IGFBP-3 serum concentrations were determined using commercially available kits (Immulite 2000, Siemens, Los Angeles, CA). All samples were tested twice in one assay, with IGF-I and IGFBP-3 having intra-assay coefficients of variation of 2.77% and 2.60%, respectively.

### Food Diary

A validated food diary was used to characterize the sample in the baseline period (time 1). Each participant had their own form and spent the day recording foods, portion sizes, and eating times. The food diaries were used on 3 alternate days over a week of everyday activities (time 1). Each individual’s caloric need, basal metabolic rate, and total energy need were also indirectly stipulated. Caloric need was estimated according to the formula of Harris and Benedict (1918).^15^ Total energy expenditure was calculated considering a very intense physical activity level (1.9), and dietary intake was calculated with the help of the TACO (Brazilian Food Composition Table).^16^

During the military field training (4 days), all the soldiers ate in the same way, at exactly the same times and with the same food portions. It was not possible to calculate the quantity of calories consumed in this period, as the concept of “real life” was respected, where the soldiers spent 4 days of training in the field without any contact with the outsiders (other than their superiors). The quantity of calories ingested was not monitored in the week after the end of the military field training (time 3). Water was supplied ad libitum during the military field training.

### Body Composition

Anthropometric evaluations (height and mass) were conducted using an electronic scale (Toledo®, São Bernardo do Campo, SP), model 2098pp, with 100 g and 0.1 cm accuracy.

The skinfold measurements were carried out using a plicometer (Cescorf®, Porto Alegre, RS; 0.1 mm accuracy). The body composition calculation used to determine the male soldiers’ body fat was carried out using the equation proposed by Jackson and Pollock (1978). Subsequently, the body fat percentage was obtained using the Siri equation.^17^ The average of 3 measurements was used in the calculations.

### Statistical Analysis

The data were analyzed using descriptive statistics (mean, standard deviation, maximum, and minimum). Because of the sample size, nonparametric statistics were used to compare the means. For the primary measurements of the study (IGF-I and IGFBP-3), the Friedman test was used to evaluate the difference in serum concentrations at the 3 data collection times. Friedman’s two-way ANOVA was used as a post hoc test to compare the measurements in pairs (times 1 and 2; 1 and 3; 2 and 3). The significance level adopted was 0.05. All the analyses were conducted in the SPSS software (Statistical Package for the Social Sciences for Windows®, IBM®, Austin, TX), version 20.0.

## RESULTS

Table I shows the sample’s descriptive data, anthropometric variations, and changes in serum concentrations of IGF-I and IGFBP-3 at the 3 data collection times.

**TABLE I.** Descriptive Data, Anthropometric Variations, and Serum Concentrations of IGF-I and IGFBP-3

		Time 1		Time 2		Time 3	
Sex	Variable	Mean	SD	Mean	SD	Mean	SD
Male (*n* = 12)	Body weight (kg)	71.54	12.83	70.86	12.74	–	–
	% body fat	9.63	4.05	9.34	3.89	–	–
	IGF-I (ng/mL)	189.67	28.98	162.6	36.62	199.17	24.04
	IGFBP-3 (ng/mL)	4.72	0.64	4.09	0.74	4.96	0.68

The results are presented as means and SDs.

Abbreviations: IGF-1, insulin-like growth factor type I; IGFBP-3, insulin-like growth factor binding protein type 3; SD, standard deviation.

The Friedman test showed that the concentrations of IGF-I and IGFBP-3 varied between the 3 data collection times (X^2^(2) = 13.915, *P* = .001; X^2^(2) = 12.500, *P* = .002; respectively), that is, a significant drop from time 1 (baseline—IGF-I: 189 ng/mL and IGFBP-3: 4.71 mg/L) to time 2 (immediately after military training—IGF-I: 162 ng/mL and IGFBP-3: 4.08 mg/L) and subsequent recovery of these markers, with a significant increase from time 2 (immediately after military training) to time 3 (a week after military training—IGF-I: 199 ng/mL and IGFBP-3: 4.98 mg/L). The post hoc analysis for Friedman’s ANOVA test showed that the concentrations of IGF-I and IGFBP-3 at time 2 differed from those collected at times 1 and 3 (*P* = .01; *P* = .07, respectively).

**Figures 1.**
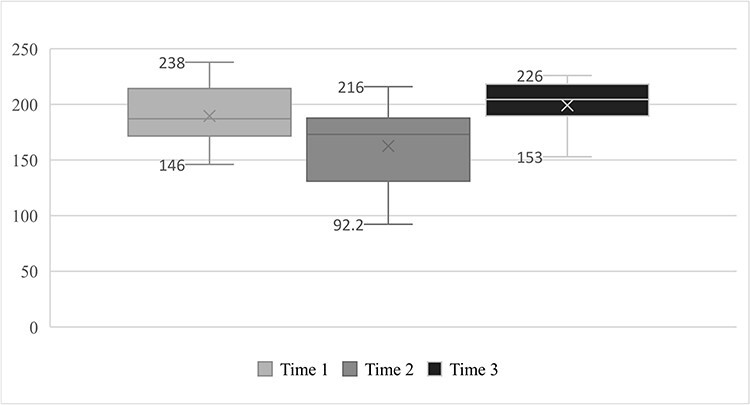
IGF-I serum levels before, during, and after field exercise training.

**Figure 2.**
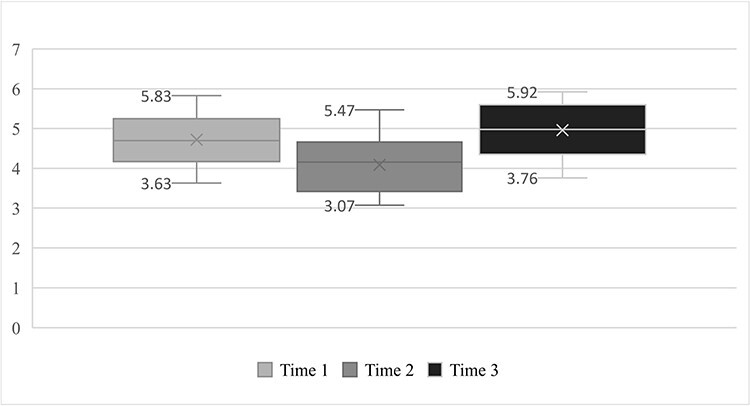
IGFBP-3 serum levels before, during, and after field exercise training.

Next (Figures 1 and 2), the IGF-I and IGFBP-3 results are presented in a line graph showing the biphasic behavior of these components.

*Indicates a significant reduction between times 1 and 2.#Indicates a significant increase between times 2 and 3.

As shown in Table I, the body composition data presented very discrete changes between time 1 (baseline) and time 2 (after military training), with the mean values and their respective standard deviations being: weight at time 1: 71.5 ± 12.2 kg and weight at time 2: 70.8 ± 12.2 kg; body fat percentage at time 1: 9.6 ± 3.8% and body fat percentage at time 2: 9.3 ± 3.7%.

Finally, the results from the food diaries used at time 1 (baseline) to evaluate each soldier’s intake are presented. Unfortunately, 3 soldiers handed their diaries in blank; because of this, there are only 9 soldiers’ data in the Supplemental Material section.

## DISCUSSION

This study shows that, despite the short period evaluated (4 days of military field training), significant responses of both IGF-I and IGFBP-3 were observed—since body composition acted in a very discrete way from time 1 (baseline) to time 2 (post-training). This fact reinforces the role of IGF-I in the rapid response to acute stressors and as a biomarker of training status.^7^ The decrease in IGF-I levels is a warning even before other detrimental responses occur (possibly significant decreases in body composition).^18^ In this study, changes in the field exercise routine were not proposed. Instead, an important practical finding was made. The routine weeks of the soldier’s training course include activities and programs that enable IGF-I and IGFBP-3 to recover to their baseline values after the specific field exercises; this was the main result.

This study reinforces the thesis that IGF-I is a metabolic marker in a short time period, primarily because of its response to an energy deficit. Other studies in field training exercises have reported similar results in short periods (4-8 days), when there is usually a drop in IGF-I and IGFBP-3^7^ and an accentuated increase in IGFBP-1.^6,8,9,11^

What few studies have observed is the recovery of these markers. Military training seeks a high-stress level, which cannot be altered, as it is an objective of the proposed activity.^14^ As a result, the endocrine part of the soldier plays a synergetic role in the body to enable strenuous training activities. Evaluating the recovery of hormones and binding proteins after the end of military operations may be essential. It could be a focus for monitoring the health of military personnel engaged in operational activities. However, the logistics involved in blood tests may be a limitation for using IGF-I and IGFBP-3 as biomarkers in field exercises.^7^

Concerning the biphasic outcomes of the markers, this was a theoretical hypothesis initially proposed by Eliakim and Nemet,^19–21^ which was replicated for the first time in the study conducted by Tourinho et al.^22^ However, the study, conducted by the same research group as that of the present article, ended up reproducing this biphasic design for the first time in swimming, with athletes in different training periods: in the specific phase of the preparatory period, IGF-I and IGFBP-3 were low, and subsequently, in the polishing phase—a load reduction period to reduce pre-competition physiological and psychological stress and achieve optimal performance levels^23^—it was possible to verify an increase in IGF-I and IGFBP-3. Although the swimming results are similar to those of the present article, our research group hypothesizes that the reasons for these responses differ.

The responses regarding reduced IGF-I and IGFBP-3 in the military environment appear to portray suppression of this hormone and its main binding protein because of the high-calorie expenditure and controlled food intake. On the other hand, in swimming, in athletes trained to their highest level for competition, who prioritize the correct distribution of training loads throughout their preparation and eat correctly, there emerges the hypothesis that serum IGF-I decreases because of more excellent tissue absorption of this hormone, accompanied by a drop in the IGFBP-3 binding protein, perhaps because of the proteolysis process of the ternary complex, thus making IGF-I more available for the tissues.

The logic behind the possible differences in the behavior of IGF-I and IGFBP-3 between conventional athletes and soldiers may be that physical activity is not structured to reach peak performance but only to simulate an extreme situation of using biomotor skills in a war situation (field exercises or simulated tasks).^24^

As shown in another study by our research group, the soldier’s sex does not seem to influence the response of the IGF-I/IGFBP-3 system. In jungle survival training, there was a significant decrease in IGF-I and IGFBP-3 concentrations in both sexes.^7^ However, studies on these differences are scarce in the literature, and since the Brazilian Air Force has no female soldiers, the sample in this study was made up of male soldiers only.

Hence, it seems reasonable to propose 2 distinct mechanisms for the biphasic behavior of this axis: one involving the effective suppression of the hormone and the other centered around tissue absorption, resulting in a decline in serum levels in both scenarios. We believe that future studies are needed to evaluate the behavior of the systemic and local responses of IGF-I to distinguish the real reason for its fall in serum in response to the stimuli applied by both physical and military training.

## CONCLUSION

IGF-I and IGFBP-3 levels respond quickly to the stimuli caused by military training, especially after specific field training (high energy expenditure and restricted diet). However, these markers quickly return to their baseline values after this type of training, simply by following the daily routine of the battalion during control weeks, without requiring any specific interventions. This fact confirms the importance of the results of this study, since the rapid return to baseline values of the IGF-I/IGFBP-3 system after an effort such as that of the Brazilian Air Force recruits indicates that the recruits can be used in new missions or operations.

## Supplementary Material

usae097_Supp

## Data Availability

The data that support the findings of this study are available on request from the corresponding author. All data are freely accessible.

## INSTITUTIONAL CLEARANCE

Institutional clearance was approved.

## References

[1] Nindl BC, Barnes BR, Alemany JA, Frykman PN, Shippee RL, Friedl KE: Physiological consequences of U.S. Army Ranger training. Med Sci Sports Exerc 2007; 39(8): 1380–7.doi: 10.1249/MSS.0b013e318067e2f7 17762372

[2] Nindl BC, Friedl KE, Frykman PN, Marchitelli LJ, Shippee RL, Patton JF: Physical performance and metabolic recovery among lean, healthy men following a prolonged energy deficit. Int J Sports Med 1997; 18(5): 317–24.doi: 10.1055/s-2007-972640 9298770

[3] Friedl KE, Moore RJ, Hoyt RW, Marchitelli LJ, Martinez-Lopez LE, Askew EW: Endocrine markers of semistarvation in healthy lean men in a multistressor environment. J Appl Physiol (1985) 2000; 88(5): 1820–30.doi: 10.1152/jappl.2000.88.5.1820 10797147

[4] Fortes MB, Diment BC, Greeves JP, Casey A, Izard R, Walsh NP: Effects of a daily mixed nutritional supplement on physical performance, body composition, and circulating anabolic hormones during 8 weeks of arduous military training. Appl Physiol Nutr Metab 2011; 36(6): 967–75.doi: 10.1139/h11-124 22111592

[5] Gomez-Merino D, Chennaoui M, Drogou C, Guezennec CY: Influence of energy deficiency on the insulin-like growth factor I axis in a military training program. Horm Metab Res 2004; 36(7): 506–11.doi: 10.1055/s-2004-825730 15305236

[6] Nindl BC, Alemany JA, Kellogg MD, et al: Utility of circulating IGF-I as a biomarker for assessing body composition changes in men during periods of high physical activity superimposed upon energy and sleep restriction. J Appl Physiol (1985) 2007; 103(1): 340–6.doi: 10.1152/japplphysiol.01321.2006 17412783

[7] Magraner JMPDS, Talarico Neto T, Hahns Júnior HC, Tourinho Filho H, Martinelli Júnior CE: Serum hormone concentrations and body composition in Brazilian Air Force cadets during rainforest survival training. Mil Med 2023; 188(11–12): 3302–08.doi: 10.1093/milmed/usac201 35803739

[8] Nindl BC, Rarick KR, Castellani JW, et al: Altered secretion of growth hormone and luteinizing hormone after 84 h of sustained physical exertion superimposed on caloric and sleep restriction. J Appl Physiol (1985) 2006; 100(1): 120–8.doi: 10.1152/japplphysiol.01415.2004 16141374

[9] Alemany JA, Nindl BC, Kellogg MD, Tharion WJ, Young AJ, Montain SJ: Effects of dietary protein content on IGF-I, testosterone, and body composition during 8 days of severe energy deficit and arduous physical activity. J Appl Physiol (1985) 2008; 105(1): 58–64.doi: 10.1152/japplphysiol.00005.2008 18450989

[10] Martinelli CE Jr, Custódio RJ, Aguiar-Oliveira MH: Fisiologia do eixo GH-sistema IGF [Physiology of the GH-IGF axis]. Arq Bras Endocrinol Metabol 2008; 52(5): 717–25.doi: 10.1590/s0004-27302008000500002 18797577

[11] Nindl BC, Castellani JW, Young AJ, et al: Differential responses of IGF-I molecular complexes to military operational field training. J Appl Physiol (1985) 2003; 95(3): 1083–9.doi: 10.1152/japplphysiol.01148.2002 12909598

[12] Clemmons DR, Klibanski A, Underwood LE, et al: Reduction of plasma immunoreactive somatomedin C during fasting in humans. J Clin Endocrinol Metab 1981; 53(6): 1247–50.doi: 10.1210/jcem-53-6-1247 7197688

[13] Frystyk J, Delhanty PJ, Skjaerbaek C, Baxter RC: Changes in the circulating IGF system during short-term fasting and refeeding in rats. Am J Physiol 1999; 277(2): E245–52.doi: 10.1152/ajpendo.1999.277.2.E245 10444419

[14] Brasil da CA : ICA 37-738 Currículo Mínimo do Curso de Formação de Oficiais de Infantaria. 2019.

[15] Harris JA, Benedict FG: A biometric study of human basal metabolism. Proc Natl Acad Sci U S A 1918; 4(12): 370–3.doi: 10.1073/pnas.4.12.370 16576330 PMC1091498

[16] Universidade Estadual de Campinas : *Tabela Brasileira de Composição de Alimentos—TACO*, 4ª edn. Unicamp; 2011.

[17] Jackson AS, Pollock ML: Generalized equations for predicting body density of men. Br J Nutr 1978; 40(3): 497–504.doi: 10.1079/bjn19780152 718832

[18] Nindl BC : Insulin-like growth factor-I as a candidate metabolic biomarker: military relevance and future directions for measurement. J Diabetes Sci Technol 2009; 3(2): 371–6.doi: 10.1177/193229680900300220 20144370 PMC2771506

[19] Eliakim A, Brasel JA, Mohan S, Wong WL, Cooper DM: Increased physical activity and the growth hormone-IGF-I axis in adolescent males. Am J Physiol 1998; 275(1): R308–14.doi: 10.1152/ajpregu.1998.275.1.R308 9688993

[20] Nemet D, Eliakim A: Growth hormone-insulin-like growth factor-1 and inflammatory response to a single exercise bout in children and adolescents. Med Sport Sci 2010; 55: 141–55.doi: 10.1159/000321978 20956866

[21] Eliakim A, Nemet D: Exercise training, physical fitness and the growth hormone-insulin-like growth factor-1 axis and cytokine balance. Med Sport Sci 2010; 55: 128–40.doi: 10.1159/000321977 20956865

[22] Tourinho Filho H, Pires M, Puggina EF, et al: IGFBP-3 and ALS concentrations and physical performance in young swimmers during a training season. Growth Horm IGF Res 2017; 32: 49–54.doi: 10.1016/j.ghir.2016.12.004 28011098

[23] Mujika I, Padilla S: Detraining: loss of training-induced physiological and performance adaptations. Part I. Sports Med 2000; 30(2): 79–87.doi: 10.2165/00007256-200030020-00002 10966148

[24] Scofield DE, Kardouni JR: The tactical athlete: a product of twenty-first century strength and conditioning. Strength Cond J 2015; 37(4): 2–7.doi: 10.1519/SSC.0000000000000149

