# Supplementary material for: Biphasic Kinetics of IGF-I and IGFBP-3 in Response to Military Field Training in Brazilian Air Force Recruits
Source: Mil Med. 2024 Apr 30;189(7-8):e1417–22. doi: 10.1093/milmed/usae097 (PMC11221556; doi:10.1093/milmed/usae097)
Supplement: usae097_Supp [file usae097_supp.zip › supplementary.doc]

**Supplementary Material –** Soldiers’ caloric need and mean intake of calories and macronutrients from three days of dietary intake

| **Soldier** | **Nº1** | **Nº2** | **Nº3** | **Nº4** | **Nº5** | **Nº6** | **Nº7** | **Nº8** | **Nº9** |
| --- | --- | --- | --- | --- | --- | --- | --- | --- | --- |
| **Age (years)** | 19 | 19 | 19 | 19 | 19 | 19 | 19 | 18 | 19 |
| **Height (cm)** | 183 | 183 | 174 | 175 | 178 | 173 | 177 | 165 | 181 |
| **Weight (kg)** | 71.5 | 72.6 | 72.3 | 72.6 | 71.6 | 74.9 | 73.7 | 57.8 | 96.6 |
| **BMR (kcal)** | 1831 | 1846 | 1797 | 1806 | 1808 | 1828 | 1831 | 1560 | 2165 |
| **TEN (kcal)** | 3480 | 3508 | 3415 | 3432 | 3435 | 3473 | 3480 | 2965 | 4114 |
| **Calorie intake (kcal)** | 1335 | 1257 | 2333 | 1355 | 1850 | 2975 | 3890 | 2843 | 2230 |
| **CHO intake(g – g/kg)** | 197 – 2.8 | 194 – 2.7 | 234 – 3.2 | 184 – 2.5 | 245– 3.4 | 414 – 5.5 | 462 – 6.3 | 321 – 5.6 | 254 – 2.6 |
| **LIP intake (g – g/kg)** | 24 – 0.3 | 30 – 0.4 | 85 – 1.2 | 28 – 0.4 | 57 – 0.8 | 155 – 2.1 | 113 – 1.5 | 130 – 2.3 | 82 – 0.9 |
| **PTN intake (g – g/kg)** | 78 – 1.0 | 54 – 0.7 | 162 – 2.2 | 89 – 1.2 | 99 – 1.4 | 133 – 1.8 | 96 – 1.3 | 97 – 1.7 | 113 – 1.2 |

**Observation:** Caloric need estimated according to the Harris and Benedict (1919) formula. TEN was calculated considering a very intense physical activity level (1.9) and dietary intake was calculated with the help of the TACO table. **Legend:** BMR – basal metabolic rate; TEN – total energy need; CHO – carbohydrate; LIP – lipid; PTN – protein.
